# Deployment and Allocation Strategy for MEC Nodes in Complex Multi-Terminal Scenarios

**DOI:** 10.3390/s22186719

**Published:** 2022-09-06

**Authors:** Danyang Li, Yuxing Mao, Xueshuo Chen, Jian Li, Siyang Liu

**Affiliations:** 1 State Key Laboratory of Power Transmission Equipment and System Security and New Technology, Chongqing University, Chongqing 400044, China; 2 Electric Power Research Institute, Yunnan Power Grid Co., Ltd., Yundaxilu, Kunming 650217, China

**Keywords:** Internet of Things, mobile edge computing, edge node deployment, genetic algorithm

## Abstract

Mobile edge computing (MEC) has become an effective solution for insufficient computing and communication problems for the Internet of Things (IoT) applications due to its rich computing resources on the edge side. In multi-terminal scenarios, the deployment scheme of edge nodes has an important impact on system performance and has become an essential issue in end–edge–cloud architecture. In this article, we consider specific factors, such as spatial location, power supply, and urgency requirements of terminals, with respect to building an evaluation model to solve the allocation problem. An evaluation model based on reward, energy consumption, and cost factors is proposed. The genetic algorithm is applied to determine the optimal edge node deployment and allocation strategies. Moreover, we compare the proposed method with the *k*-means and ant colony algorithms. The results show that the obtained strategies achieve good evaluation results under problem constraints. Furthermore, we conduct comparison tests with different attributes to further test the performance of the proposed method.

## 1. Introduction

The fifth-generation mobile communication system (5G) provides the opportunity for more devices to access the IoT, and a considerable amount of terminal devices and services designed to meet user needs is emerging, resulting in a massive increase in the volume of data required for services accessing wireless networks [1]. The way tasks are uploaded to the cloud for calculation solves the problem of a large number of services and generated tasks to be calculated; however, due to the limitation of transmission distance, this solution is not quite applicable to latency-sensitive task devices. The emergence of MEC makes it possible to perform resource-intensive and delay-sensitive device tasks, especially in machine learning and artificial intelligence [2,3] improving user experience [4]. By sinking computational resources closer to the terminal devices, MEC not only allows for a more flexible allocation of computational resources [5,6] but also data isolation from the cloud, improving the stability of the device and network connections [7].

A large number of existing studies have considered edge nodes [8,9], but the location of the edge node is directly fixed, and the impact of the edge node location on task processing and computation offloading is ignored [10]. However, in contrast to cloud computing, the computational resources of MEC are limited by energy and resource costs. In several deployment scenarios, the appropriate location cannot be determined directly as a matter of course.

**Figure 1:**
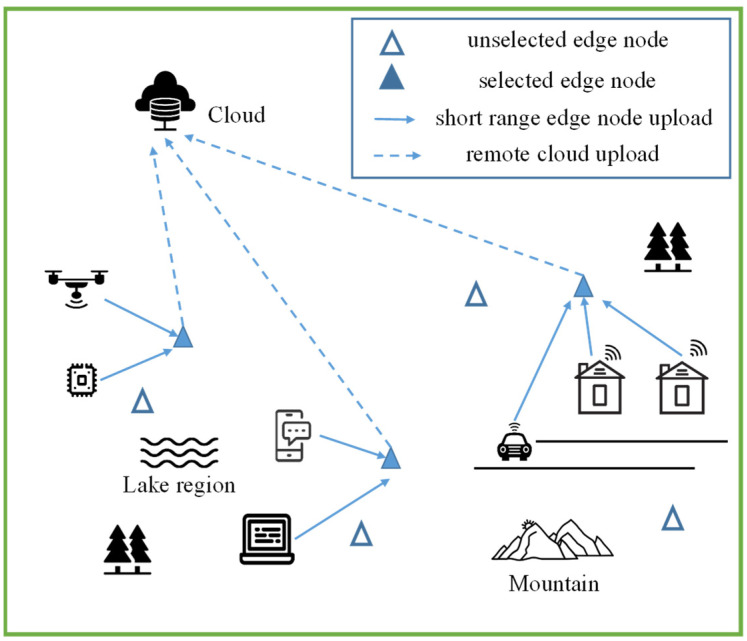
The architecture of multiple terminals, edge nodes, and clouds.

Appropriate locations for edge nodes should be selected based on local topographical conditions and power supply conditions. In extreme cases, lakes and mountains are not suitable for node placement, as shown in Figure 1. Solid triangles are represent edge nodes for data processing, whereas hollow triangles are not selected. Datasets are uploaded from terminals to edge nodes, and the processing results are uploaded to the could. Moreover, the location differences in edge nodes directly determine the distance between edge nodes and terminals, and the distribution also affects the utilization of computing resources, which further affects latency and transmission energy consumption [11,12]. If the edge nodes cannot be reasonably selected for allocation, energy is wasted, and the time delay caused by unreasonable nodes seriously affects the processing of real-time tasks. Therefore, selecting an appropriate location for edge nodes and allocating terminal tasks is the basic condition for making full use of computing resources and improving the performance of the entire edge computing system. Furthermore, the strategy of location deployment selection should satisfy the task processing requirements in scenarios with different parameters.

In this paper, we focus on the problem of edge node deployment and computational resource allocation for complex terminals. Here, we obtain the solution to the optimization function with three influencing factors (including processing reward, energy consumption, and deployment cost) by comparing three evolutionary optimization algorithms, obtaining the optimal deployment allocation strategy. The main contributions of the present study are as follow:

- We design a model for complex terminal application scenarios. By analyzing the characteristics of terminal emergency and power supply modes, the model is suitable for specific application scenarios. We consider the processing capability of edge nodes and ultimately select suitable nodes among several potential edge nodes.
- An improved genetic algorithm (GA) is designed and compared with the *k*-means and ant colony (ACO) algorithms. We analyze the advantages and disadvantages of the three algorithms in terms of the iterative process, task assignment balance, and final optimization results. GA achieves the best performance; therefore, it is used to test different terminal attributes.
- We test the performance of the algorithm under different scenarios and analyze its applicability under varying terminal and edge node sparsity conditions, task flow rates, data volume ranges, and task processing complexities, providing a reference for subsequent application studies to explore additional scenarios.

The rest of this paper is arranged as follows. In Section 2, we present the mainstream studies and research progress associated with current deployment problems. In Section 3, we describes specific problems and the model-building process. In Section 4, we illustrate three algorithms for model solving. In Section 5, we describe the selection of parameters and analysis of the distribution and deployment results. Finally, conclusions are provided in Section 6.

## 2. Literature Review

Several studies have been conducted on edge node deployment in different application scenarios, such as vehicular networking and industrial design. Vehicular networking uses edge computing applications to process and store vehicle data. Jabri et al. [13] used an ant colony optimization algorithm to select gateways and the number of connected fog nodes to improve the utilization of gateways and nodes in both static and dynamic mobility scenarios. Chang et al. [14] constructed a multi-objective optimization model to characterize the tradeoffs between three key performance metrics: initial deployment cost, run time cost, and average delay of the task, decomposing the problem using a heuristic multi-objective optimization approach.

Industrial applications are focused on data processing of highly sensitive devices in smart factories and smart manufacturing scenarios. Jiang et al. [15] applied an improved *k*-means clustering algorithm to the deployment of edge nodes in smart manufacturing systems by synthetically balancing the optimization objectives of network latency, computational resource deployment cost, and edge node computational capacity. Wang [16] established a model to optimize the deployment cost and load balancing, proposing a fault-tolerant server deployment scheme and improving the global optimal solution of the algorithm using a binary-based gray wolf genetic policy algorithm.

The deployment of edge nodes involves the influence of many factors, and many studies have considered the impact of deployment allocation according to the application. Cao et al. [17] considered the impact of heterogeneous edge servers and the fairness of base station response latency on user quality of service. The authors proposed optimization methods for the offline and online phases to achieve joint optimization of the expected response delay of the system and the base station based on fairness criteria. Lin [18] minimized the total installation cost using a metaheuristic algorithm combined with a discrete monkey algorithm, considering the constraints of maximum demand capacity, maximum delay time, coverage area, and maximum equipment capacity. Jia et al [19] studied cloudlet placement and terminal allocation to cloudlets in a wireless metropolitan area network (WMAN). They proposed a density-based clustering (DBC) algorithm to solve the NP hard problem. Zhang [10] designed a particle swarm optimization algorithm for an edge cloud placement strategy with a focus on minimizing the total time cost of task execution. Luo [20] proposed a deep q-learning and reinforcement learning algorithm that models the problem as a Markov decision process and uses reinforcement learning to obtain optimal placement results based on latency. Mann [21] proposed a decentralized allocation strategy to improve overall flexibility, creating fog clusters for devices in different regions and establishing connections between clusters for transmission. Herrera [22] studied the application of software-defined networking (SDN) in edge computing scenarios and analyzed the deployment of nodes in SDN Internet topology and industrial IoT infrastructures using heuristic methods [23].

Based on our literature review, we conclude that current research is focused on solving problems using different algorithms and testing the adaptability of the algorithms in different scenarios. However, existing studies do not consider the extended properties of terminals, i.e., the task urgency and the power supply mode. Analytical comparisons between current research and our work are presented in Table 1. The task urgency set can guarantee the resource tendency in the urgent processing of some tasks, whereas other authors focus on the power supply mode to keep the system running for a longer time. “√” indicates that the corresponding factor is considered, and “×” indicates that the factor is not considered.

## 3. System Model

In this section, we analyze the edge node deployment problem and describe the investigated scenario. To obtain the solution set for edge node selection, we design a multi-objective optimization function to measure the performance of edge node deployment based on the three components of concern: reward, energy consumption, and deployment cost. Finally, latency and data volume balance are added as effects to measure the optimization function.

### 3.1. Scenario Description

**Figure 2:**
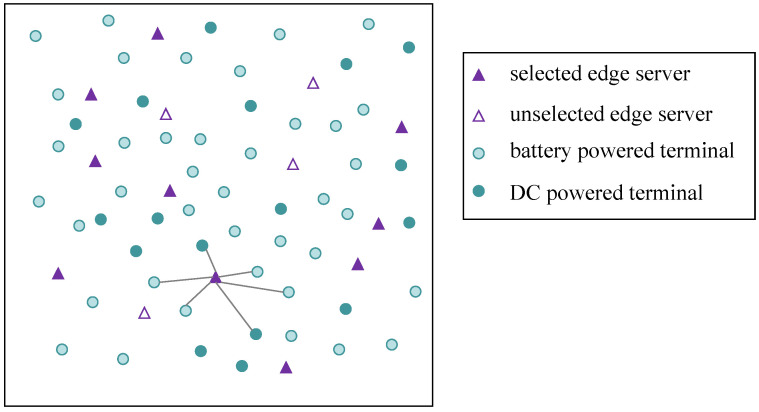
Terminal and edge node distribution diagram.

A square area is established in which IoT terminal devices are located, and terminal devices and potential edge nodes are randomly distributed, as shown in Figure 2. We assume that the wireless network has *n* terminal devices, denoted by $n_{i}\{i=1,2,\cdots ,n\}$, whereas there are m potential edge nodes for selection, denoted by $m_{j}\{j=1,2,\cdots ,m\}$. The *n_i_* features are represented as $N_{i}=\{x_{i},y_{i},D_{i},\partial_{i},C_{i},\lambda_{i},P_{i}\}$, $N=\{N_{1},\ldots ,N_{i},\ldots ,N_{n}\}$.

- Urgency $\partial_{i}$: In the actual scenario, different locations correspond to terminals with different requirements for time tolerance sensitivity, so we divide terminals into three levels of urgency $\partial_{i}=\{1,2,3\}$, with $\partial_{i}=3$ as the most urgent terminal and $\partial_{i}=1$ as the least urgent terminal. During processing, tasks created by high-urgency terminals have a preference with respect to the degree of reward, as specifically described in Section 3.2.2.

- Task complexity $C_{i}$: Task complexity affects the amount of CPU time spent on task processing. The higher the complexity of the task, the longer the processing time. For example, edge nodes consume more computational resources than text processing when dealing with image-type tasks. In this paper, we consider four common task types: ASCII compression, data table reading, variable-bit-rate (VBR) coding, and constant-bit-rate (CBR) coding, with a complexity of y of 330 cycles/byte, 960 cycles/byte, 1300 cycles/byte, and 1900 cycles/byte, respectively [24].

- Task rate $\lambda_{i}$: indicates the number of tasks generated by terminal i per unit of time, where the task-generated rate ($\lambda_{i}$) by terminals obeys the Poisson distribution.

- Power supply modes $P_{i}$: Each terminal device has two power supply modes, i.e., battery-powered (harsh environment, unable to meet the conditions of the external power supply) or direct power supply. In particular, only one of the two power supply modes is available for each terminal. Thus, the power supply method of the terminal can be expressed as Equation (1). $P_{i}=P_{b}$ means that the terminal is powered by a battery, whereas $P_{i}=P_{d}$ means that the terminal is powered by DC. $P_{b}$ and $P_{d}$ indicate the energy consumption weights of the two power supply modes. In Figure 2, the dark green and light green circles represent different power supply modes of terminals.

(1) $$P_{i}=\{\begin{array}{cc} P_{b} & \mathrm{battery}-\mathrm{powered}\\ P_{d} & DC-\mathrm{powered} \end{array}$$

The feature set for $m_{j}$ is represented as $M_{j}=\{x_{j},y_{j},C_{j},d_{j\max},D_{j\max}\}$, $M=\{M_{1},\ldots ,M_{j},\ldots ,M_{m}\}$. $\left\{x_{j},y_{j}\right\}$ indicates location information of edge nodes, $C_{j}$ indicates the processing performance of the edge node in processing tasks, $d_{ij\max}$ indicates the farthest wireless transmission distance between edge nodes and terminals (distance between $n_{i}$ and $m_{j}$ is presented as $d_{ij}$), and $D_{j\max}$ indicates the maximum amount of data that the node can receive per unit time. Due to the limitations of power supply and special terrain conditions, only *m* potential location points in the square area satisfy the possibility of becoming edge computing nodes. After the selection of potential nodes, the final number of placed edge nodes is obtained and set as *k*.

The connection matrix of edge nodes and terminals is represented as Equation (2). If $O_{ij}=1$, *n_i_* is connected to $m_{j}$; otherwise, $O_{ij}=0$.

(2) $$O=\left[\begin{array}{ccc} O_{11} & \cdots & O_{n1}\\ \vdots & O_{ij} & \vdots \\ O_{1m} & \cdots & O_{nm} \end{array}\right]$$

In the remainder of this section, we display the optimization function to evaluate the merits of deployment, as well as the calculation methods of latency, energy consumption, and cost factors.

### 3.2. Optimization Function Design

To measure the optimal function of different edge node selections and allocations, three factors are included: the reward function that an edge node can receive to successfully transmit and process tasks, the impact of transmission on energy consumption cost in terms of power supply mode, and the varying cost required to deploy varying numbers of edge nodes. 

#### 3.2.1. Optimization Function

The function associated with deploying edge nodes uses a reward–cost structure; therefore, we use profit to represent the function, integrating the impact of deployment cost, processing completion reward, and energy consumption cost on edge node selection and allocation.

(3) $$\max:F_{pro}=\frac{F_{reward}{}^{\alpha }}{F_{power}{}^{\beta }F_{cost}{}^{\gamma }}$$

(3a) $$\sum_{j=1}^{m}O_{ij}=1$$

(3b) $$\sum_{i=1}^{n}O_{ij}D_{i}\leq D_{j\max}$$

(3c) $$d_{ij}<d_{ij\max}$$

(3d) $$\mu_{j}>\frac{1}{T_{j}}$$

The function $F_{pro}$ consists of three parts: the value of the reward ($F_{reward}$), energy consumption ($F_{power}$), and deployment cost ($F_{cost}$). α, β, and γ are the exponential elements for each of the three parts. Our aim is for the reward value to increase and the energy and cost to decrease, so $F_{power}$ and $F_{cost}$ are set as the denominator, and $F_{reward}$ is set as the numerator. 

The constraints are explained as follows. All the terminals need to be and can only be connected to one edge node, which avoids the situation of missed or multiple connections (3a). The total amount of data volume received by each edge node per unit of time should be less than or equal to its maximum carrying data volume, which ensures the balance of task distribution and protects the carrying capacity of the edge nodes (3b). With respect to the communication distance constraint between edge nodes and terminals (3c), when the task load is too heavy and queues are blocked, the allocation fails to meet the task requirements, so such cases should be directly removed from the deployment strategies, and the variables ($\mu_{j}$) are expressed in Equation (12) (3d). Details of the three-part function are described below.

#### 3.2.2. Influencing Factor of Reward

The whole system is rewarded by completing tasks. Thus, the task completion reward function is determined by the latency ($t_{i}$) of task transmission, queuing, and processing. Terminals with different urgency requirements have different latency sensitivities, so the function for each urgency is as follows.

(4) $$F_{reward}=\{\begin{array}{c} \sum_{i=1}^{n}c_{r}O_{ij}\lambda_{i}(\sqrt{\partial_{i}s}-\partial_{i}t_{i})(t_{i}\leq t_{i0})\\ 0\;\;\;\;\;\;\;\;\;\;\;\;\;\;\;\;\;\;\;\;\;\;\;\;\;\;\;\;\;\;\;\;\;\;\;\;\;\;\;(t_{i}>t_{i0}) \end{array}$$

The area of the reward function for different urgency degrees is a constant value (S), that is, the product of the task time and the reward value is a constant value in order to ensure the fairness of the rewards obtained for tasks with different degrees of urgency, which leads to Equation (4). The task has a maximum latency limit ($t_{i_{0}}$), which is the intersection of the function with the horizontal axis.

(5) $$t_{i0}=\sqrt{\frac{s}{\partial_{i}}}$$

If the total latency ($t_{i}$) of the task is less than $t_{i_{0}}$, the corresponding reward is obtained, whereas if $t_{i}$ exceeds $t_{i_{0}}$, no reward is be obtained. Tasks with high urgency requirements have an appropriately higher degree of reward, and latency is more tolerated for low-urgency tasks.

**Figure 3:**
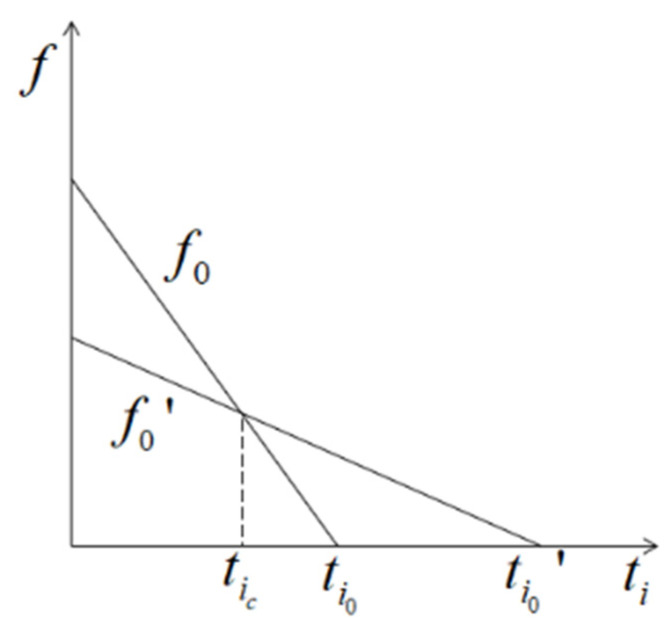
The relationship between reward function and latency.

An example of different urgencies of the reward function is shown in Figure 3, which demonstrating the reward function of urgency $\partial_{0}$ and ${\partial_{0}}^{\prime }$ when $O_{ij}$ and $\lambda_{i}$ are fixed. The function $f_{0}$ has a higher urgency than that of function ${f_{0}}^{\prime }$. The horizontal coordinate of the intersection of the two functions is $t_{i_{c}}$. Along the horizontal coordinate, as the latency increases, the values of the reward functions $f_{0}$ and ${f_{0}}^{\prime }$ continuously decrease. If $t_{i}\in (0,t_{i_{c}}]$, $f_{0}$ has a higher reward value. If $t_{i}\in (t_{i_{c}},t_{i_{0}}]$, low-urgency tasks have greater tolerance for latency, and the reward value (${f_{0}}^{\prime }$) is higher compared to $f_{0}$. If $t_{i}\in (t_{i_{0}},{t_{i_{0}}}^{\prime }]$, $f_{0}=0$, indicating that the reward for the higher-urgency function ($f_{0}$) could not be obtained, and the reward value of the low-urgency function (${f_{0}}^{\prime }$) decreases until it reaches the maximum tolerance latency (${t_{i_{0}}}^{\prime }$).

(6) $$t_{i}=t_{Ti}+t_{Qi}+t_{Pi}+t_{Ri}$$

The calculation of latency includes the time of task transmission ($t_{Ti}$), task queuing ($t_{Qi}$), processing time ($t_{Pi}$), and data return ($t_{Ri}$). Due to the low data volume of data return, $t_{Ri}$ is negligible and omitted here, compared with other latency.

1. Transmission latency $t_{Ti}$

The transmission time of tasks is determined by data volume ($D_{i}$) and wireless communication transmission rate ($R_{ij}$). The transmission rate between terminals and edge nodes can be obtained according to Shannon’s theorem as follows.

(7) $$T_{Ti}=\frac{D_{i}}{R_{ij}}$$

(8) $$R_{ij}=w_{ij}\log_{2}(1+\frac{P_{tx}H_{i}}{\sigma^{2}})$$

In Equation (8), $w_{ij}$ denotes the communication bandwidth, $P_{tx}$ denotes the transmission power of terminals, and $\frac{P_{tx}H_{i}}{\sigma^{2}}$ is the signal-to-noise ratio of the uplink channel. The average channel gain ($H_{i}$) follows the free-space path loss model.

(9) $$H_{i}=A_{d}{\left(\frac{3\times 10^{8}}{4\pi f_{c}d_{ij}}\right)}^{de}$$

(10) $$d_{ij}=\sqrt{{\left(x_{i}-x_{j}\right)}^{2}+{\left(y_{i}-y_{j}\right)}^{2}}$$

where $H_{i}$ denotes the antenna gain, $f_{c}$ denotes the carrier frequency, de denotes the path loss index, and $d_{ij}$ is the Euclidean distance between $n_{i}$ and $m_{j}$.

2. Queuing latency $t_{Qi}$

The task rate received by $m_{j}$ gathering from the terminals that are connected with $m_{j}$, shown as $\lambda_{j}$, is obtained through the tasks generated by $n_{i}$ per unit of time.

(11) $$\lambda_{j}=\sum_{i=1}^{n}O_{ij}\lambda_{i}$$

(12) $$\mu_{j}=\frac{1}{\frac{\sum_{i=1}^{n}O_{ij}t_{Pi}\lambda_{i}}{\sum_{i=1}^{n}O_{ij}\lambda_{i}}}$$

The maximum number of tasks ($\mu_{j}$) that a node can process per unit time is calculated according to the time required to process a unit number of tasks, expressed as $\frac{\sum_{i=1}^{n}O_{ij}t_{Pi}\lambda_{i}}{\sum_{i=1}^{n}O_{ij}\lambda_{i}}$.

(13) $$t_{Qi}=\frac{1}{\mu_{j}-\lambda_{j}}-\frac{1}{\mu_{j}}=\frac{\lambda_{j}}{(\mu_{j}-\lambda_{j})\mu_{j}}$$

The difference between the time a task stays at an edge node $\frac{1}{\mu_{j}-\lambda_{j}}$ and the time it takes for the task to be processed ($\frac{1}{\mu_{j}}$) is the queuing time ($t_{Qi}$) required for the task to arrive.

3. Processing latency $t_{Pi}$

(14) $$t_{Pi}=\frac{C_{i}}{C_{j}}$$

The processing time ($t_{Pi}$) is determined by the complexity of the task ($C_{i}$) generated by $n_{i}$ and the processing capability ($C_{j}$) of $m_{j}$.

#### 3.2.3. Influencing Factor of Battery Power

The energy consumption factor is related to the power supply mode of terminals. In order to ensure a longer service life of the battery-powered terminal, we emphasize the energy consumption value of the battery-powered terminals.

(15) $$F_{power}=\sum_{i=1}^{n}c_{p}P_{i}\lambda_{i}E_{i}$$

The influence factor of consuming unit energy is denoted as $c_{p}$. The transmission distance within the set range of energy consumption is available in the free space model, and the distance factor has the following effect on energy loss [25].

(16) $$E_{i}=D_{i}E_{elec}+D_{i}\xi_{fc}d_{ij}{}^{2}$$

where $E_{elec}$ denotes the energy consumed per unit of data received and transmitted, and $\xi_{fc}$ is the energy consumed per unit of data transmitted per unit length of the transmitting amplifier.

#### 3.2.4. Influencing Factor of Deployment Cost 

The number of edge nodes to be deployed is constrained by the value of the deployment cost.

(17) $$F_{c}=kc_{c}$$

The actual number of deployed edge nodes (k) indicates that the amount of edge nodes determines the cost, and $c_{c}$ indicates the cost associated with deployment per unit edge node.

#### 3.2.5. Performance Metrics

To fully characterize the results of edge node allocation, we select two values that have received attention in task processing and computation to aid the objective function in measuring the merit of the function.

1. Latency

Latency is generated during the resolution of each terminal task, and the latency of each task is aggregated to reflect the overall processing sensitivity of the system.

(18) $$latency=\sum_{i=1}^{n}T_{i}$$

2. Deployment balance

The balance of deployment has a considerable impact on the stability of the system, so the variance of data volume is used as a measure of deployment balance.

(19) $$B_{j}=\sum_{i=1}^{n}O_{ij}D_{i}\lambda_{i}$$

(20) $$Variance=\sqrt{\frac{\sum_{j=1}^{k}{\left(B_{j}-\bar{B}\right)}^{2}}{k}}$$

When deployed, the total amount of data ($B_{j}$) received by $m_{j}$ is the product of the amount of data ($D_{i}$) per task and the task-generated rate ($\lambda_{i}$). The value of Variance is an important measure of the algorithm when several algorithms are compared.

## 4. Description of Algorithms

To obtain the best edge node selection and allocation strategy, we use three evolutionary optimization algorithms: *k*-means, ACO, and GA. In this section, we describe, in detail, the solution concepts of the three algorithms applied to a complex scenario.

### 4.1. k-Means

*k*-means is a cluster analysis algorithm for fast cluster resolution based on the distance value of each point from the cluster center [26]. The *k*-means algorithm requires specification of the size of *k* values in advance, so we designed the selection of *k*, which is determined by the data volume ($D_{j\max}$) received by $m_{j}$.

(21) $$k\geq \frac{\sum_{i=1}^{n}D_{i}}{D_{j\max}}$$

The *k* value must be chosen to satisfy the limit of the maximum capacity of data volume and minimize the deployment cost of the device with reasonable settings. Terminals are divided into subclusters, and each subcluster has an edge node for task processing. We define the whole set of subclusters ($C_{k}$) as $C(C_{k}\in C)$. The cluster center (pk),which is located in $L_{pk}(x_{pk},y_{pk})$, in a single subcluster ($C_{k}$), is the center of all terminals in the subcluster ($C_{k}$) by distance. The terminals in the subcluster ($C_{k}$) are defined as $n_{k}$, and the total number of terminal locations is $n_{c}$; an update of $L_{pk}(x_{pk},y_{pk})$ is shown in the following equations.

(22) $$L_{pk}(x_{pk},y_{pk})=\frac{\left(\sum_{nk=1}^{n_{c}}x_{nk},\sum_{nk=1}^{n_{c}}y_{nk}\right)}{n_{c}}$$

(23) $$d_{i,pk}=\sqrt{(x_{i}-x_{pk}{)}^{2}+{\left(y_{i}-y_{pk}\right)}^{2}}$$

The updated cluster center location is calculated by aggregating all the node location information within the cluster, as shown in Equation (22). Then, the Euclidean distance ($d_{i,pk}$) from the terminal to each center is recalculated, and the nearest cluster center is selected for the terminal until the position of the center no longer changes, and the final cluster center ($pk_{0}$) is obtained.

(24) $$d_{j,pk_{0}}=\sqrt{(x_{j}-x_{pk_{0}}{)}^{2}+{\left(y_{j}-y_{pk_{0}}\right)}^{2}}$$

After obtaining the subclusters and their centers ($pk_{0}$), the potential node with the shortest distance ($d_{j,pk_{0}}$) between $m_{j}$ and $pk_{0}$ is selected as the edge node for each subcluster. The connection between the terminal device and the edge node is derived from the subcluster, from which the final edge node selection and terminal assignment are directly derived. The pseudocode of *k*-means is shown in Algorithm 1.
**Algorithm 1: *k*-means for the Placement Scheme**Input: M, N, Number of cluster kOutput: O, $F_{pro}$
Randomly select *k* points as the starting center$C_{k}=\emptyset$Do: Set $C_{k}$ by $\min:d_{i,pk}(x_{pk},y_{pk})$
While $L_{pk}$ changes$L_{pk}\leftarrow L_{pk_{0}}$Find $O_{ij}$ by minimal $d_{j,pk_{0}}(x,y)$
Calculate $F_{pro}$


### 4.2. ACO

**Figure 4:**
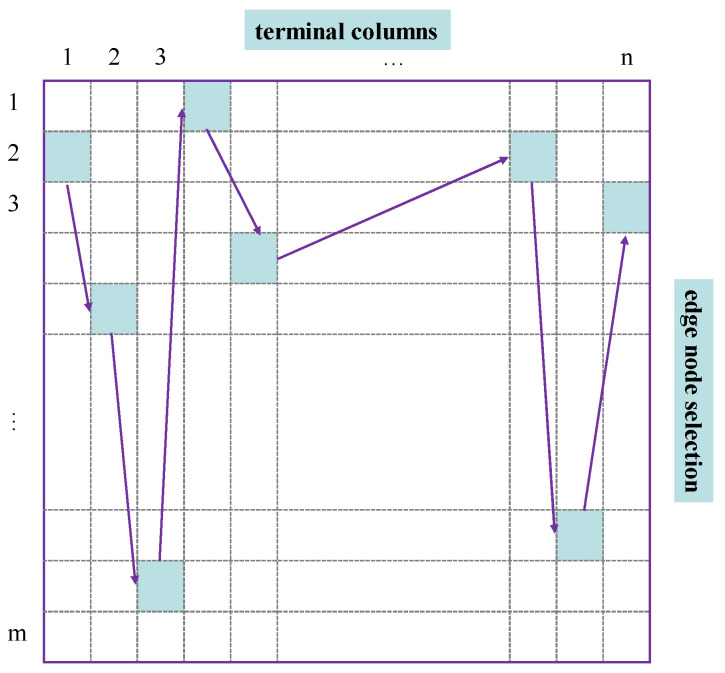
One search path grid of ACO.

The ACO is a method to optimize the goal problem by simulating an ant path search using the pheromone while searching for a specific goal [27]. The ant search path is set as a combination of the selected edge node numbers for each terminal; therefore, the path can be expressed in an m×n grid, in which the ant searches for the best path, as shown in Figure 4. Each column represents the selection range of one terminal, and the ant searches each column from left to right to obtain the edge node for each terminal. In Figure 4, the $n_{a}$ path is $n_{1}$ terminal connected to edge node $m_{2}$, $n_{2}$ terminal connected to edge node $m_{5}$, and go forward by column until the search path ($Pa_{n_{a}}=\{2,5,m-1,\cdots ,4\}$) is obtained.

(25) $$T=\left[\begin{array}{ccc} \tau_{1,1} & \cdots & \tau_{1,n}\\ \vdots & \tau_{j,i} & \vdots \\ \tau_{m,1} & \cdots & \tau_{m,n} \end{array}\right]$$

The pheromone matrix (T) records the pheromone intensity of connection between edge nodes and terminals, indicating the pheromone value of the grid in row j and column i. 

Based on the current pheromone matrix, the probability ($p_{ij}(t)$) of an ant selecting different edge nodes for terminal $n_{i}$ in iteration t can be calculated as:

(26) $$p_{ij}(t)=\frac{\tau_{j,i}^{\alpha_{ph}}(t)}{\sum_{j=1}^{m}\tau_{j,i}^{\alpha_{ph}}(t)}$$

The information factor ($\alpha_{ph}$) indicates the degree to which the ant’s path selection is influenced by the pheromone. There are a total of Na ants, and the ant is defined as $n_{a}$. The path ($Pa_{n_{a}}(t)$) is selected via roulette wheel selection to generate a new ant, from which the connection matrix (O(t)) can be obtained. The value of the objective function under such a selection strategy is obtained from the connection matrix, which is defined as $F_{pro}{}^{n_{a}}(t)$.

(27) $$\Delta \tau_{j,i}(t)=\{\begin{array}{cc} \sum_{n_{a}=1}^{N_{a}}F_{pro}{}^{n_{a}}(t) & \left(O_{ij}(t)=1\right)\\ 0 & \left(O_{ij}(t)=0\right) \end{array}$$

(28) $$\tau_{j,i}(t+1)=(1-\rho )\tau_{j,i}(t)+\Delta \tau_{j,i}(t)$$

The new pheromone ($\tau_{j,i}(t+1)$) is obtained based on the value of the function, which means the difference is the updated value of the pheromone ($\Delta \tau_{j,i}(t)$). ρ is the volatilization factor of the pheromone. ACO updates the pheromone intensity matrix (T) and the probability ($p_{ij}(t)$) to provide a reference for the path selection of ants in the subsequent generations. After multiple generations of ant evolution, the pheromone update enables the paths of ants to converge to an optimal route, at which time the best allocation and deployment scheme is obtained. The pseudocode of the ACO is shown in Algorithm 2.
**Algorithm 2: ACO for the Placement Scheme**Input: M, N
Output: O, $F_{pro}$
$n_{a}\leftarrow 0$, $Pa_{N_{a}\times n}=\emptyset$While $n_{a}<N_{a}$
Initial $Pa_{n_{a}}$
$Pa_{N_{a}\times n}\leftarrow Pa_{N_{a}\times n}\cup Pa_{n_{a}}$$n_{a}\leftarrow n_{a}+1$EndWhile $t<N_{r}$
Foreach $Pa_{n_{a}}(t)$ doCreate $Pa_{n_{a}}(t)$ by $p_{ij}(t)$
Get $O_{ij}(t)$ &$F_{pro}(t)$
$\tau_{j,i}(t)\leftarrow \tau_{j,i}(t+1)$Endt←t+1EndFind $O_{ij}$ by $\tau_{j,i}$
Calculate $F_{pro}$


### 4.3. GA

GA simulates the principle of generation of individuals in natural biological populations, an optimization algorithm that uses cross-mutation rules similar to those of genes to generate quality individuals [28]. There is a total of $N_{p}$ individuals in the population, and the final dominant individual is obtained after the population is updated for $N_{r}$ iterations based on the crossover probability ($P_{c}$) and mutation probability ($P_{m}$). We set the r individual in the population that is a deployment scheme individual $G_{r}=\{g_{r,1},g_{r,2},\cdots ,g_{r,n}\}$: the length of the individual gene is n, indicating that n terminals are required to select their corresponding edge nodes, and the value of each gene is chosen in the range {1,2,3,…,m}, which corresponds to the total set of potential edge nodes.

Edge nodes and the corresponding connections between terminals and edge nodes (O) can be directly selected from specific individuals, followed by calculation of the function value. The individuals in the population must satisfy the constraints of the problem: the connection distance cannot exceed the maximum communication requirement, and the amount of received data cannot exceed the maximum capacity of the edge node. If the newly generated individuals do not satisfy the constraints, then the cross-variation operation should be performed again until individual sets reach the population size. The process of GA is divided into four parts.

1. Selection of parents

To select suitable parents, roulette wheel selection is used to screen individuals. The fitness of individuals ($p_{r}$) in a population determines the probability that an individual can produce offspring, with the most fit individuals being more likely to produce offspring and less fit individuals less likely to produce offspring.

(29) $$p_{r}=\frac{F_{pro}{}^{r}}{\sum_{r=1}^{N_{r}}F_{pro}{}^{r}}$$

We set the fitness as the function ($F_{pro}{}^{r}$) for each individual and obtain the fitness probability of selecting r individuals for the crossover variation as the probability ($p_{r}$) that individuals with greater fitness are more likely to be selected as parents. The probability of crossover ($P_{c}$) determines the number of new individuals generated by each population crossover, and the number of new individuals is $N_{r}P_{c}$. Therefore, in one iteration, there are $\frac{N_{r}P_{c}}{2}$ selection processes, and the individuals that have already been selected will not be selected in the next parent selection.

2. Crossing

**Figure 5:**
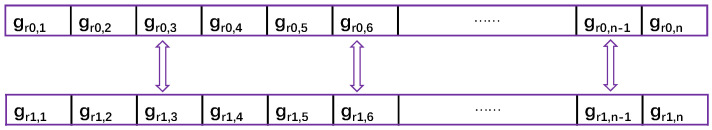
Paternal gene crossover process.

To improve the efficiency of offspring diversification, the crossover strategy involves the selection of three genes for exchange in each crossover operation. For instance, as shown in Figure 5, the third, sixth, and (n−1)th genes are selected.

3. Mutation

Because the possibility of mutation-generating gene change during the process of population update improves the diversification of offspring, we design the mutation strategy to decide whether new individuals generate mutation according to the mutation probability value ($P_{m}$). After determining the individuals to be mutated, a gene is randomly selected, and one edge node is selected to replace the former.

4. Generation of new populations

To save the dominant individuals of the population, the number of $N_{r}(1-P_{c})$ dominant individuals from the original population for the new population is selected. The new population retains $N_{r}$ individuals, which ensures that the total number of individuals in the population does not change from generation to generation.

The pseudocode of the GA is shown in Algorithm 3.
**Algorithm 3: GA for the Placement Scheme**Input: M, N
Output: O, $F_{pro}$
$n_{a}\leftarrow 0$, $G_{n_{gene}\times n}=\emptyset$ ${G_{n_{gene}\times n}}^{\prime }=\emptyset$While $n_{gene}<N_{p}$
Initial $G_{n_{gene}}$
$G_{n_{gene}\times n}\leftarrow G_{n_{gene}\times n}\cup G_{n_{gene}}$$n_{gene}\leftarrow n_{gene}+1$EndWhile $t<N_{r}$
Sort $G_{n_{gene}\times n}(t)$ by $F_{pro}(t)$
While parents $<N_{r}P_{c}$ doSelect parents $G_{n_{0}}(t)$ &$G_{n_{1}}(t)$ by $p_{r}$
Apply crossing to get offspring $G_{n_{0}}(t+1)$ &$G_{n_{1}}(t+1)$
Apply mutation to $G_{n_{0}}(t+1)$ &$G_{n_{1}}(t+1)$
$G_{n_{gene}\times n}(t+1)\leftarrow G_{n_{gene}\times n}(t+1)\cup G_{n_{0}}(t+1)\cup G_{n_{1}}(t+1)$parents←parents+2EndSort $G_{n_{gene}\times n}(t)$ for finding $G_{N_{r}(1-P_{c})}(t)$
$G_{n_{gene}\times n}(t+1)\leftarrow G_{n_{gene}\times n}(t+1)\cup G_{N_{r}(1-P_{c})}(t)$$G_{n_{gene}\times n}\leftarrow G_{n_{gene}\times n}(t+1)$t←t+1EndFind $O_{ij}$ by $G_{n_{gene}\times n}$
Calculate $F_{pro}$


## 5. Results

### 5.1. Experimental Conditions

All simulation experiments described in this section are performed on a PC equipped with an i5-11400 CPU and 16 GB RAM, and all algorithms are implemented in MATLAB (R2016a, MathWorks. Inc., Natick, MA, USA). The simulation experiments are set up in a 1000 × 1000 m^2^ IoT square, and the initial number of terminals and potential edge nodes is set to *n* = 50 and *m* = 25, respectively. The specific parameters of the optimization function are shown in Table 2, and the parameters of the algorithms are illustrated in Table 3.

To simplify the model and adapt to the needs of a variety of scenarios, it is necessary to set some initial rules for the scenario. The location of devices within the scenario is fixed, and the location of potential edge nodes is determined by the preliminary site inspection; we use specific points to delegate potential locations, and these points are also set to a fixed location. We set the complexity of terminal tasks within a particular scenario to a fixed value, that is, the complexity of a terminal task is uniquely determined, and the complexity of the generated task changes as the scenario changes. To meet the processing requirements of all scenarios, the storage resources of the edge nodes are set to accommodate all application services. The communication parameters are set as Ad=4.11, $f_{c}=915\;\mathrm{MHz}$, de=2.8 [29]. $E_{elec}=50\;\mathrm{nJ}/\mathrm{bit}$, and ξ=10 pJ/bit [30].

### 5.2. Results Analysis

In this section, we present an analysis and discussion of the algorithm comparison. First, in Section 5.2.1, we analyze and compare the performance of the three investigated algorithms according to reference factors: energy consumption, data distribution balance, and deployment cost. The genetic algorithm achieves the best performance; therefore, we change the relevant key evaluation indices under different parameter settings, which are tested in Section 5.2.2.

#### 5.2.1. Comparison of Three Algorithms

**Figure 6:**
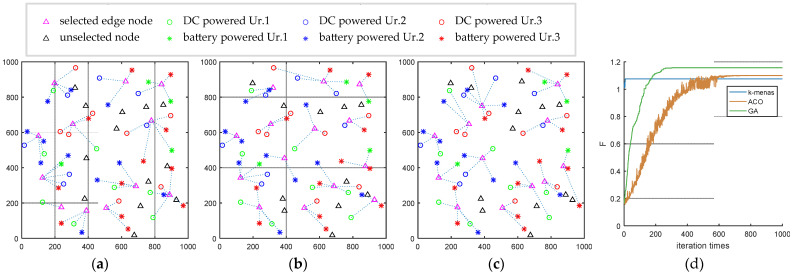
(**a**) *K*-means distribution; (**b**) ACO distribution; (**c**) GA distribution; (**d**) iteration comparison.

The comparison experiments of the three algorithms are conducted with the processing task of 960 cycles/byte and the computing processing performance of the edge nodes at 10 GHz. (a), (b), and (c) in Figure 6 show the deployment planes of the three algorithms. The characteristics of terminals are identified by power supply and urgency. The power supply mode is distinguished by icon shape; the DC power supply is presented as a hollow circle, whereas the battery power supply represented by is a quincunx mark. Urgency is marked by color, and terminals with an urgency of 1, 2, and 3 are indicated by green, blue, and red, respectively. Potential edge node locations are indicated by hollow triangles, and whether they are selected is distinguished using color, with pink indicating selected and black indicating unselected.

The result of the *k*-means algorithm has a *k* value of 12, and the number of terminals connected to each edge node varies between one and six; the ACO results in 14 edge nodes, and the terminals connected to each edge node varies between two and five; the GA results in 12 edge nodes, and the number of terminal devices connected to each edge node vary between three and seven. In the *k*-means algorithm, the number of connected edge nodes varies within a wide range, and connecting only one terminal causes the computational resources of the nodes to be wasted, whereas the number of connected nodes with the GA and ACO span a narrow range with appropriate clusters. The process of algorithm convergence is shown in Figure 6d. *K*-means searches for the final value within 20 generations, ACO converges to the best deployment result after around 600 generations, and GA converges after around 300 generations. GA obtains the best function value after convergence.

A parameter comparison of the three algorithms is shown in Table 4. The three algorithms obtain relatively consistent reward parameter values, with the ACO having the highest reward value, GA with the second highest reward value, and *k*-means with the lowest reward value. The *k*-means algorithm obtains the highest value for the energy consumption component, and the ACO consumes the least energy. The ACO selects 14 edge nodes, resulting in higher deployment cost compared to the GA and *k*-means models. The final total objective function value of the GA is the highest, which means that the best deployment result can be obtained by combining the three partial parameters of reward, energy consumption, and the number of nodes. The GA algorithm has the lowest variance value of clustering and the best data balance, which means the possibility of blockage is reduced, and system allocation is improved. The *k*-means algorithm only considers the single distance factor, but obtains a clustering result faster with a computation time of 0.883 s. Due to the lack of other factors, the performance of the *k*-means algorithm is not sufficient in terms of energy consumption and data balance. The computation time of the ACO is 46.574 s; however, a longer computation time is not conducive to deployment. Combining various factors, we conclude that the GA achieves the best performance and the best convergence effects.

#### 5.2.2. Four Parameter Changes for the Investigated Scenario

To observe the performance of the deployment strategy under different scenarios when the device and edge node parameters are changed, we design the following four sets of experiments. According to the solution of described in Section 5.2.1, GA is used for simulation.

1. Changing the number of edge nodes and terminals

**Figure 7:**
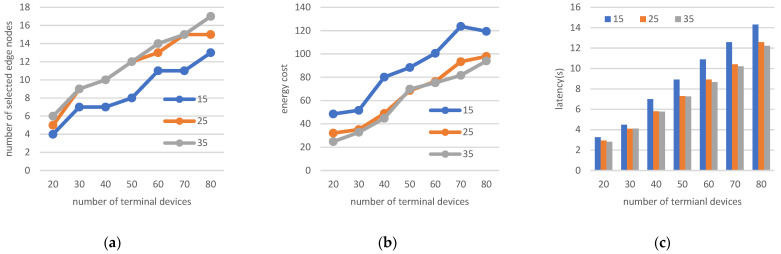
(**a**) Number of selected edge nodes; (**b**) energy cost; (**c**) latency when changing the number of edge nodes and terminals.

For this experiment, the terminal task complexity is set as $C_{i}=960\;\mathrm{cycles}/\mathrm{byte}$, and node computational power is set to 10 GHz. The number of terminals varies between 20 and 80, and analysis is performed for cases with 15, 25, and 35 potential edge nodes. The obtained experimental results are shown in Figure 7.

The change in the number of terminals influences the sparsity of the scenario, whereas an increase in the number of potential edge nodes results in more selections of potential nodes. As shown in Figure 7a, the processing requirement of computing resources forces the number of selected nodes to increase if there are more terminals; hence, the number of selected edge nodes increases by an average factor of one during the increase in the number of terminals from 20 to 80. As the number of selected nodes increases, the distance between terminals and nodes decreases, leading to mitigation of the energy consumption shown in Figure 7b and the latency of data presented in Figure 7c. As shown in Figure 7c, as the number of terminals increases, the time consumption increases from about 3 s to 12–14 s. In the case of fixed terminals, the number of potential edge nodes varies by 15, 25, and 35; the latency gradually decreases; and the decrease in latency of potential nodes from the blue line (15 potential edge nodes) to the orange line (25 potential edge nodes) is more obvious, whereas latency is not obvious when the number edge nodes varies from 25 to 35. This indicates that researchers should focus on the number of potential nodes and provide enough potential edge nodes for calculation processing. In the process of increasing the number of terminals from 20 to 80, the distribution of terminals gradually becomes denser, requiring more edge nodes and consuming more energy. In a dense terminal scenario, providing more potential locations for edge nodes effectively reduces the delay of the whole system and saves resources by reducing energy consumption.

2. Changing the number of terminals and node processing capacity

**Figure 8:**
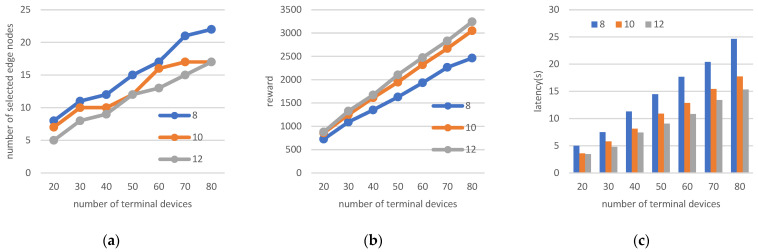
(**a**) Number of selected edge nodes: (**b**) energy cost; (**c**) latency when changing the number of terminals and node processing capacity.

We set the data volume to 1–1.5 MB, the task frequency to 0.8/s, the terminal task complexity to $C_{i}=960\;\mathrm{cycles}/\mathrm{byte}$, and the number of potential edge nodes to m = 25. The performance and parameters of the system are analyzed when the number of terminals varies between 20 and 80, and the processing performance of potential edge nodes is 8 GHz, 10 GHz, and 12 GHz. The obtained results are shown in Figure 8.

The larger the number of terminals, the more edge nodes are selected to meet the demand for computing resources under the condition that the processing performance of potential edge nodes remains unchanged. When the potential edge node processing performance increases from 8 GHz to 12 GHz, the number of edge nodes required decreases, as indicated by the blue line passing through the yellow line to the gray line in Figure 8a. The increase in the number of terminals causes the number of processing tasks to increase exponentially; hence, the total reward value obtained by completing tasks increases by 2.4 times from 20 to 80 terminals in Figure 8b with a processing capacity of 8 GHz. Furthermore, as the processing capacity of the edge node increases, the reward value obtained in the case of the same number of terminal devices also gradually increases, as indicated by the lines in the figure. An increase in processing capability can significantly reduce the latency, as shown in Figure 8c. When the node capability is increased from 8 GHz to 10 GHz with 80 terminals, the latency time decreases by 28.0% and by 13.6% when the processing capability is increased from 10 GHz to 12 GHz.

3. Changing the amount of data and generated task rate

**Figure 9:**
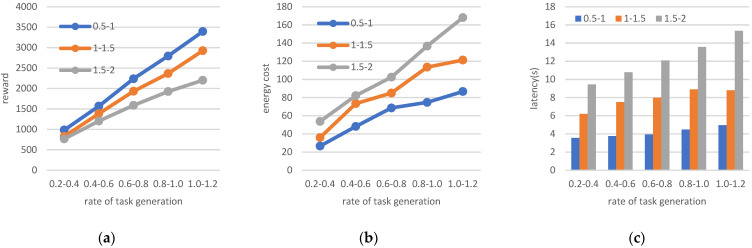
(**a**) Reward; (**b**) energy cost; (**c**) latency when changing the amount of data and task rate.

The initial values set for this experiment are *n* = 50, *m* = 25, $C_{i}=960\;\mathrm{cycles}/\mathrm{byte}$, and $C_{j}=10\;\mathrm{GHz}$. We analyze the performance of the system when the frequency of terminal-generated tasks varies between 0.2/s and 1.2/s and the amount of task-generated data ranges are 0.5–1 MB, 1–1.5 MB, and 1.5–2 MB. The results are shown in Figure 9.

With a constant range of data generated by the task, the higher the frequency of the task generated by the terminals, the more energy consumed to meet the demand of the transmission, as shown in Figure 9b. For the data range of 1.5–2 MB, the1.0–1.2/s rate increases energy consumption by 1.13 times compared to the 0.2–0.4/s rate. The latency increases in Figure 9c, and the obtained reward value increases, as shown in Figure 9a, with an increased rate of task generation. As shown in Figure 9a, for tasks in the range of 1.5–2 MB (the gray line), the 1.0–1.2 MB/s rate increases the reward value by a factor of 1.87 compared to a rate of 0.2–0.4 MB/s. When the generated data ranges from 0.5–1 MB to 1.5–2 MB, the time required for transmission increases and affects the reward value function, as shown by the blue line passing through the yellow line to the gray line in Figure 9a, indicating a decrease in the reward value, suggesting that the limitations of the transmission require that the end task be generated in such a way that the amount of data transmitted is compressed as much as possible to obtain an improved reward value for the tasks while satisfying all the information requirements for the calculation.

**Figure 10:**
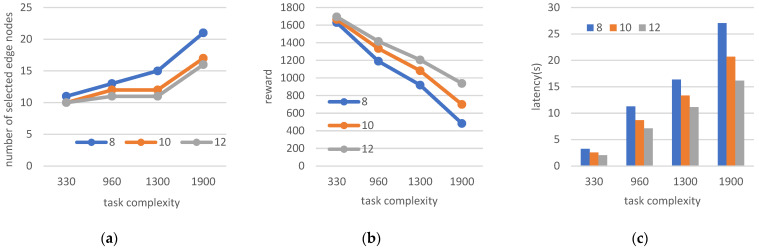
(**a**) Number of selected edge nodes; (**b**) reward; (**c**) latency when changing the task complexity and edge node processing capability.

4. Changing task complexity and edge node processing capability;

The initial values set for this experiment are n = 50 and m = 25, with the data volume of the terminal task in the range of 1–1.5 MB, and a task-generated rate of 0.8/s. The performance and parameters of the system are analyzed when the processing complexity of the terminal-generated tasks are 330, 960, 1300, and 1900 and the processing performance of the potential edge nodes is 8 GHz, 10 GHz, and 12 GHz. The obtained experimental results are shown in Figure 10.

With respect to the constant processing performance of the potential edge nodes, the higher the complexity of the task generated by the terminal, the higher the demand for computing resources of the edge nodes. When the processing capacity is constant, e.g., the 8 GHz blue line in Figure 10a, the number of nodes selected gradually increases from 11 to 21 when the task complexity increases from 330 cycle/type to 1900 cycle/type. When the potential edge node processing performance increases from 8 GHz to 12 GHz in the process, the number of edge nodes required decreases, as shown by the blue line passing through the yellow line to the gray line in Figure 10a. Similar to changing experiment 2, increasing the computational power can reduce the number of edge nodes selected and save the cost of the number of deployed edge nodes. The more complex the task, the longer the time required to queue and process the task, as shown in Figure 10c; the total latency increases as the complexity of the horizontal coordinate increases. Furthermore, enhancement of the processing capability of the edge nodes can significantly reduce the latency time of the system; when the node capability is increased from 8 GHz to 10 GHz, the latency significantly decreases. For example, when the processing complexity of the task is 1900 cycle/type, the latency time decreases by 23.5%, whereas when the node capability is increased from 10 GHz to 12 GHz, the latency time decreases by 21.9%. As shown in Figure 10b, due to the influence of increased latency, the reward reduces with the increased task complexity when the other parameters are fixed. As the processing capacity of the edge node increases, the reward value obtained in the case of the same number of end devices is gradually increased, as indicated by the difference between the three lines.

## 6. Conclusions and Future Work

The deployment allocation problem of edge nodes has an important impact on the flexible design of edge computing components and the improvement of resource and energy utilization efficiency. In this paper, we propose a measurement method for deployment allocation of edge nodes, integrating three influencing factors: processing reward, energy consumption, and deployment cost. We also conduct a comparison test of the three algorithms through simulation to select the best algorithm for a variety of scenarios and system performance analysis.

This work can be extended in many directions. In the future, we will mainly focus on the following aspects.

- The current complex actual deployment scenario involves more requirements with respect to the service types of edge node task processing, and we hope to add planning for the services of edge nodes in conjunction with the deployment strategy in the future.
- Using a machine learning approach to strategic allocation for edge node selection can improve efficiency and simplify planning [31].
- In response to the increase in the number of terminals and the change in data, we will introduce more parameter settings that match the actual situation, fully consider the nonlinear relationship between generated task data volume and task complexity, and design a more scientific and complete system cost model.
- Due to the variety of mobile edge node applications, subsequent work should consider a dynamic edge node deployment strategy.

## Figures and Tables

**Table 1:** Factor comparisons based on a literature review.

Reference	Terminal Attributes				Evaluations		
	Data	Task Flow Rate	Power Supply Mode	Urgency	Edge Node Number	Energy Consumption	Latency
This paper	√	√	√	√	√	√	√
[14]	√	×	×	×	√	×	√
[18]	√	√	×	×	√	×	√
[16]	√	√	√	×	√	√	√
[15]	√	√	√	×	×	√	√
[17]	√	×	×	×	√	×	√

**Table 2:** Summary settings of key notations.

Notation	Details
n	[20, 80]
m	[10, 40]
$P_{b}$	1
$P_{d}$	0.2
$\partial_{i}$	[1, 2, 3]
S	1
$\lambda_{i}$	[0.2–1.2]
$C_{i}$	[330, 960, 1300, 1900, 2100] cycles/byte
$D_{i}$	[0.5–2] MB
$D_{j_{\max}}$	8 MB
$d_{ij\max}$	500 m
$C_{j}$	[8, 10, 12] GHz
α,β,γ	1, 1, 1.3
$c_{r},c_{p},c_{c}$	50, 100, 1

**Table 3:** Summary settings of key parameters for the algorithms.

Notation	Details
$N_{r}$	1000
$N_{a}$	400
$\alpha_{ph}$	1
ρ	0.7
$N_{p}$	400
$P_{c}$	0.9
$P_{m}$	0.05

**Table 4:** Comparison of three algorithms.

Algorithm	$F_{pro}$	$F_{reward}$	$F_{power}$	Number of Edge Nodes	Latency	Run Time	Variance
*k*-means	1.075	1834.894	67.494	12	5.749	0.883	0.441
ACO	1.099	1854.426	54.580	14	5.463	46.574	0.390
GA	1.156	1842.622	63.005	12	5.637	13.918	0.282

## Data Availability

Please contact the corresponding author for available data support.
